# Associations between retinal nerve fiber layer changes in glaucoma and functional gait metrics

**DOI:** 10.1093/geroni/igaf122.3886

**Published:** 2025-12-31

**Authors:** Niranjani Nagarajan, Neil Alexander, Mark Redfern, Emma Baillargeon, Lauro Ojeda, Srinath Soundararajan, Rakie Cham, Amanda Bicket

**Affiliations:** University of Michigan, Ann Arbor, Michigan, United States; University of Michigan, Ann Arbor, Michigan, United States; University of Pittsburg, Pittsburg, Pennsylvania, United States; Northwestern University, Chicago, Illinois, United States; University of Michigan, Ann Arbor, Michigan, United States; University of Michigan, Ann Arbor, Michigan, United States; University of Pittsburg, Pittsburg, Pennsylvania, United States; University of Michigan, Ann Arbor, Michigan, United States

## Abstract

Glaucoma affects mobility measured in clinic. This work investigated associations of structural measures of glaucoma with at-home mobility, an indicator of real-world function. We recruited 38 participants with glaucoma and captured spatiotemporal gait characteristics in clinic and at home using inertial measurement units. Recent optical coherence tomography retinal nerve fiber layer (RNFL OCT) measurements – a structural severity indicator – were obtained from participants’ medical records. We performed two sets of regression analyses: In the first, each gait metric was a dependent variable and RNFL OCT an independent variable; in the second, within-subject differences between in-clinic and at-home gait were dependent variables. Thinner superior RNFL (more advanced glaucoma) in the better eye was associated with reduced step regularity (p = 0.04) and increased step duration variability (p = 0.03) in clinic. Thinner inferior RNFL in the worse eye was associated with slower gait (p = 0.05) and shorter steps (p = 0.03) at home. Additionally, thinner inferior RNFL in the worse eye was associated with slower gait (p = 0.01), shorter step length (p = 0.02), and longer step duration (p < 0.01) at home vs in-clinic. Our findings expand the common perceptions that a person’s better eye drives function in glaucoma and that mobility is most impacted by superior RNFL thinning. We find new associations of inferior RNFL thinning in the worse eye with slower gait and shorter steps at home and with greater differences between at-home and in-clinic gait. The finding that more glaucomatous worse eye may make someone susceptible to gait change in real-world environments has not been reported previously.

